# The Empathic Capacity and the Ability to Regulate It: Construction and Validation of the Empathy Management Scale (EMS)

**DOI:** 10.3390/healthcare9050587

**Published:** 2021-05-15

**Authors:** Miguel Mora-Pelegrín, Beatriz Montes-Berges, María Aranda, María Agustina Vázquez, Elena Armenteros-Martínez

**Affiliations:** 1 Department of Psychology/Social Work Faculty, University of Jaén, Paraje las Lagunillas, 23071 Jaén, Spain; mmp00020@red.ujaen.es; 2 Department of Psychology/Social Psychology Area, University of Jaén, Paraje las Lagunillas, 23071 Jaén, Spain; bmontes@ujaen.es (B.M.-B.); avm00013@red.ujaen.es (M.A.V.); 3 Department of Health Science, University of Jaén, Paraje las Lagunillas, 23071 Jaén, Spain; eam00024@red.ujaen.es

**Keywords:** empathy, empathic process, healthcare professionals, validation, emotional regulation

## Abstract

The aim of this study was to develop a measure to evaluate the management of empathic capacity. To this end, two studies were conducted. Study 1 (*N* = 277, 172 females) describes the scale creation procedure, factorial validity, and internal consistency. The exploratory factor analysis yielded a five-factor model with 18 items (62.4% of the variance explained). The dimensions were as follows: D1: identification, D2: incorporation, D3: reverberation, D4: separation, and D5: projection. The internal consistency was good (alpha values ranging from 0.70 to 0.80). Study 2 (*N* = 480, 323 females) examined the validity (including convergent validity) of the model and the relationships with sociodemographic variables. The five-factor model showed a robust goodness of fit, χ^2^ = 240.5, *p* < 0.001, root mean square residual (RMSR) = 0.05. The fit indices were satisfactory, Non-normed fit index (NNFI) = 0.89, comparative fit index (CFI) = 0.90, mean square error of approximation (RMSEA) = 0.04. The convergent validity analysis showed that, as empathy management increased, so too did the empathy level and emotional intelligence. Some differences by age and sex were found. In conclusion, the Empathy Management Scale is a valid and reliable instrument for analyzing the empathic process that allows vulnerabilities and strengths to be estimated, which could improve professional practice in the healthcare context.

## 1. Introduction

Over the past few decades, empathy has been widely studied in the context of medicine, nursing, and other healthcare professions. Empathy is necessary for successful professional–user interactions [1]; it also plays a relevant role in therapeutic relationships and the quality of care [2,3,4]. In addition, empathy is related to personal welfare (e.g., preventing burnout in healthcare professionals) [5].

Most scientific studies have focused on defining empathy and explaining the underlying mechanisms and processes [6,7,8,9]. From a psychometric perspective, studies have focused on the measurement of empathy. Existing instruments mainly capture the degree of empathy that an individual has or the development among its components [10,11,12]. However, despite knowledge of the importance of the balance between being empathic and properly managing this capacity for the psychological wellbeing of professionals, specific and validated measurements on this topic are lacking.

Studies have confirmed the importance of the individual’s capacity to handle each of the stages involved in the process of empathy. When alterations in some of the empathic processes occur, such as emotional and social adaptation or regulation of the state of mind, the individual’s control over his or her behavior is reduced. This is particularly relevant for professionals who must empathize with others experiencing intense and negative emotions. When “empathy bridges the gap between self-experience and that of others” [13] (p. 19), professionals are at greater risk. Research has shown a strong relationship between empathy and feeling guilty [14,15], and difficulty in managing the stressors associated with secondary traumatic stress. Moreover, a failure of empathic regulation may lead to compassion fatigue [15]. Empathy is also considered a necessary interpersonal skill for satisfactory professional–user interactions [16,17]. However, successful use of empathy is not related to feeling it intensively, but rather to applying it in such a way that it does not cause suffering or wear down professionals [18,19,20].

Because the adaptive use of empathy is highly relevant to individual development, and due to its role in other psychological processes and applications in the field of healthcare, the present study aims to create and validate an instrument to measure the management of empathy.

### 1.1. Overview of the Empathic Process

The scientific study and conceptualization of empathy, i.e., the exploration of its nature and the processes involved, started long ago. Such work has given rise to a broad theoretical and empirical corpus, so an overview of the context in which instruments measuring empathy develop is required.

A classic study by Rogers [21] described empathy as the ability to communicate by sensing the client’s feelings as though they are the therapist’s own, but without becoming bound up by them and losing the sense of self. Here, a defining quality is the recognition of the other’s feelings “as if” they are the therapist’s own, but with awareness of the distinction. According to this proposal, empathy involves the ability to accurately perceive the internal frames of reference of others in terms of their meanings and emotional components [22]. This definition highlights that empathy is both an emotional and cognitive process. Considering empathy as a process, professional engagement only on a cognitive level could be deemed superficial; therefore, to enhance the interaction, building trust within the therapeutic relationship through emotional engagement is necessary [21,22].

These two dimensions of emotional (feeling the emotion of someone else as our own) and cognitive empathy (mentally putting oneself in someone else’s shoes) are emphasized by Davis [10]. He devised a multidimensional model, including both of these dimensions and four interconnected components There are two factors in the cognitive dimension: perspective taking, i.e., trying to understand the emotional situation of others, and fantasy, which is the cognitive tendency to imagine ourselves in the shoes of another. Regarding the emotional dimension, Davis suggested that it comprises two factors: empathic concern (orientated towards the other), defined by feelings of compassion, concern, and care when someone is feeling upset, and distress or personal unease (oriented towards oneself), which reflects the anxiety and discomfort experienced by a person when observing the negative experiences of others [6,12].

Davis’ contribution is still relevant to the multidimensional approach, and has recently been revived thanks to the neuropsychological research revealing the functional and neuroanatomical bases of emotional empathy (related to mirror neurons) and cognitive empathy (high-level cognitive functions) [7,23]. However, the relationships among these four components are not organized into stages.

Some decades before, Reik (1948) [9] in fact made such a contribution, establishing a series of sequential stages, together comprising the “empathy process”, which were necessary for the optimal management of empathy: identification, incorporation, reverberation, and separation. Identification consists of understanding and experiencing someone else’s emotions through the information transmitted by that person’s verbal and non-verbal language. It implies understanding the other’s feelings and adopting his or her perspective in order to predict what he or she thinks or feels. Each person will grasp others’ emotions to different degrees, and will vary in terms of their need for previous information. Repetto [24,25] reviewed Reik’s theory and noted that this first stage is the most relevant in the complete sequence of the empathy process. Without it, the subsequent stages cannot occur. Incorporation involves taking the other person’s experiences as our own. For Repetto, it is sometimes difficult to distinguish this from the first stage, as both constitute ways of living someone else’s experience. However, incorporation consists not only of understanding the other person’s feelings, but also experiencing them as our own. In this stage, the empathic person can take the feelings of others to such an extent that he or she might identify with the situation and feel it as if he or she were the main character. Reverberation concerns understanding the meaning of what is being expressed, and the other person’s internalized feelings are compared to the experience of such a situation. When this occurs, the person can distinguish between the emotions coming from his or herself and those that do not. Finally, in the separation stage, after the abovementioned stages have occurred, internalized emotions are reorganized and, rationally and consciously, the empathic person creates distance and is able to carry out an objective analysis and provide an appropriate response.

Thus, identification can be considered as the first prerequisite for empathy. Incorporation refers to a deepening of empathy through the addition of emotion sharing, while reverberation and separation concern the awareness of oneself that is essential for psychological wellbeing.

When a person can fulfill these phases effectively by identifying her/himself with her/his interlocutor, incorporating the interlocutor’s emotions, understanding that they belong to the other, and, finally, separating themselves while remaining at peace and in a state of wellbeing, we can say that their empathy management process is optimal. It is possible to fulfill certain stages to a greater or lesser degree; for example, a given individual may be better at reverberating and separating than at “joining up”, i.e., incorporating emotions.

These four stages also constitute abilities. Another ability crucial for empathy, but is not included in Reik’s model as a stage, is projection, i.e., the capacity to project an emotion. This ability is extensively used by healthcare professionals, although not always consciously. To transmit an air of peace, nurses may hold the patient’s hand to create a feeling inside, which is considered a valued skill in nursing [26], and is relevant to other techniques, such as therapeutic ones [27,28].

Reik’s theory, later reviewed by Repetto, was introduced decades ago. However, its tenets could be included in current approaches to empathy as a social skill, especially in the healthcare sector, to which it is particularly relevant. In this regard, an appropriate balance among the different stages of the empathy process will be reflected in the professional–client interaction, from the capacity to understand the feelings of others (identification and incorporation) to the ability to maintain a balance between sharing the person’s emotional state and ensuring enough distance to avoid overload (reverberation and separation), ultimately leading to an appropriate intervention.

### 1.2. Empathy Assessment

The wide variety of approaches to empathy has given rise to a considerable number of measuring instruments [29]. One of the first relevant scales was the Dymond Rating Test of Insight and Empathy [30]. It was designed to assess the ability to consider someone else’s perspective (the cognitive domain of empathy). Some years later, based on a unidimensional approach, the Hogan Empathy Scale was created (HES) [31]. This scale focuses on emotional components, and has been widely used to assess self-esteem, temperament, sensitivity, and non-conformism. The Mehrabian Emotional Empathy Scale (EES) [32] was designed based on unidimensional models of emotion. It assesses two aspects of empathy: the absence of aggressiveness towards others and the provision of help. The scale was subsequently revised to create the Balanced Emotional Empathy Scale (BEES) [33]. These tools are concerned with different aspects of empathy that are not sequentially related; they measure dissociable facets (such as the provision of help; the EES) or variables that influence empathy (such as sensitivity, temperament, and self-esteem; the HES) rather than the stage-related abilities required for optimal and healthy empathy.

It was not until the creation of the Interpersonal Reactivity Index (IRI) [10] that empathy began to be assessed multidimensionally. This instrument measures the four components of empathy proposed by Davis, and has been validated in Spanish in several studies [12,34]. One of the few instruments originally created in Spanish with such an integrated approach is the Test of Cognitive and Affective Empathy (Test de Empatía Cognitiva y Afectiva (TECA)) [35]. This questionnaire has four subscales: perspective adoption, emotional comprehension, empathic stress, and empathic joy. A year after its publication, a shorter version was created, the Vicarious Experience Scale (VES) [36], which introduces an assessment of the tendency towards experiencing vicarious emotional reactions. The Questionnaire of Cognitive and Affective Empathy (QCAE) [37] is another useful measure of both empathy dimensions.

The instruments described above provide information on the characteristics of empathy and its related processes from the perspective of one of its dimensions (emotional or cognitive) or both (multifactor model). They allow us to establish the level that an individual has reached for each characteristic, as well as how empathetic they are overall. The Scale of Empathic Personality (Escala de Personalidad Émpata (EPE)) [38], originally written in Spanish, measures the capacity for empathy and the ability to handle certain aspects of it. Scores on its four dimensions (feelings, ‘ápata’ traits, separation or reverberation, and simplicity) establish at which point on the empathy continuum a person is. This instrument, based on the psychoneurological model of empathy [39], adopts a multidimensional approach to the construct. However, this instrument (EPE) focuses on the personal level of empathy, rather than the stage-related abilities essential for optimum empathy management.

In the context of healthcare professionals, a systematic review was conducted on the tools used for measuring empathy in nurses [13]: the EES [32], the Barrett–Lennard Relationship Inventory (BLRI) [40], the IRI [6], the Empathy Construct Rating Scale (ECRS) [41], the BEES [33], and the Jefferson Scale of Physician Empathy (JESPE) [42]. The ECRS was the most widely used instrument with acceptable psychometric quality; the others could be improved at the theoretical or psychometric level. Yu and Kirk [43] reviewed the empathy scales applied to nursing staff, but found no “gold standard” tool.

### 1.3. Variables and Instruments Related to Empathy Management

Since the 1990s, the emotional ability necessary for interaction with other people has been studied from the perspective of emotional intelligence (EI) [44]. Mayer and Salovey’s model [45] states that EI comprises a cluster of cognitive abilities related to: (a) perceiving and expressing emotions in a precise manner, (b) using emotions to promote thinking, (c) understanding emotions, and (d) regulating emotions to enable emotional and intellectual growth. In this sense, empathy is part of the construct of EI as the ability to understand the emotions of others and handle our own through such connections in a prosocial way [46].

For assessing EI, the Trait Meta-Mood Scale (TMMS) [47] was the first self-report instrument to evaluate in a stable way over time the metaknowledge of emotional states, thus allowing the identification of individual differences in the skills required to be aware of our own emotions and the ability to regulate them [48]. It seems, therefore, that EI and empathy share some processes, particularly those related with the use, understanding, and management of emotional states in oneself and others to solve problems and regulate behaviors [49].

The large variety of approaches used to measure empathy reflects the complex nature of this construct. However, most of them focus on evaluating the level reached by a person in a particular domain of empathy, but do not specifically assess their ability to manage it. Considering that improving empathy skills is essential for nursing students [50], a measure to determine empathic ability or stage training is required. As this area has not yet been explored, the main purpose of the present work was to create a scale to measure the processes involved in the management of empathy in order to assess and help healthcare professionals balance their own feelings with those of their patients. The scale is based on multidimensional models of empathy [6,10,21], as well as Reik’s theory [9], later reviewed by Repetto [24,25], on the stages of the empathy process. Because they constitute dissociable phases and abilities, these five aspects (identification, incorporation, reverberation, separation, and projection) are measured individually. This scale is necessary to provide a means for specifically assessing ability in the different stages of empathy management. A means to evaluate these abilities is necessary to devise exercises through which they can be enhanced [51]. To this end, two studies were conducted. The first one describes the theoretical background and procedure used to create the scale, as well as its factorial validity and internal consistency. The second study was designed to examine the validity of the factorial model, the convergent validity, and the relationships with sociodemographic variables.

## 2. Study 1

### 2.1. Materials and Methods

#### 2.1.1. Participants

In total, 277 participants from Andalucía (Spain) were selected by controlled purposive sampling (62.2% female and 38.8% male). Age ranged between 18 and 49 years (M = 21.3, SD = 2.8). All participants were nursing students in their fourth year who had been honing their nursing skills in the hospital setting and had completed more than 80 h of practice when the data were collected.

#### 2.1.2. Development of Items for the Empathy Management Scale

**Figure 1:**
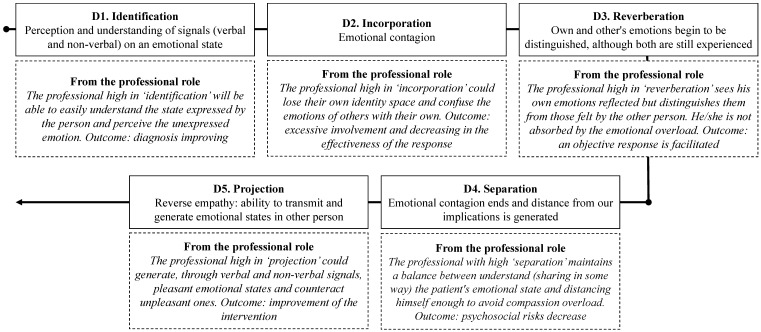
Sequence of the empathy process according to the EMS (adapted from Reik) and application to professionals.

For the creation and validation of the Empathy Management Scale (EMS), the recommendations of Muñiz and Fonseca-Pedrero [52] were followed. An initial cluster of 40 items was created. The items were based on the main theoretical underpinnings of empathy and the delimitation of the processes relevant to empathy management [9,10,21]. The relations of this construct with other variables (i.e., the capacity to feel empathy and EI) were also considered. Moreover, the content of the items was based on different aspects of the empathy processes/stages proposed by Reik [9], together with a fifth process named projection, i.e., the ability to generate emotions in others, conveying information related to a specific emotion to one or more people. This transferal takes place in most daily life situations, unconsciously and through non-verbal communication and physical interaction between two or more people [38]. This stage regulates the emotions of others. There are also items describing processes that occur automatically (e.g., the contagion of an emotion) and controlled processes that constitute actions regulated consciously (e.g., proactively preparing oneself for a difficult situation). Overall, the items comprising the five dimensions were designed to identify strengths and weaknesses in the use of empathy, and to obtain a comprehensive model on empathy management that is particularly suited for interventions (Figure 1).

The initial 40 items were screened by three experts on empathy (i.e., researchers on interpersonal processes with clinical intervention experience) who assessed their appropriateness, clarity, precision, and adequacy. Likewise, experts were requested to analyze the level of representativeness of each theoretical dimension. As a result of this process, nine items were removed due to not having an appropriateness index higher than 80%. Furthermore, the wording of four items was changed. The remaining items were considered appropriate, clear, precise, and adequate. This review resulted in a preliminary cluster of 31 items.

A five-point Likert-type scale was chosen to assess the degree of agreement or disagreement with the statements (1 = fully disagree and 5 = fully agree). In order to calculate the scores (weighted means), reverse items were inverted; higher scores indicated more effective empathy management.

#### 2.1.3. Procedure

The present study was approved by the Ethical Research with Humans Committee of the University of Jaen. Participants were provided with information about the research and their rights (objectives, identification of researchers, anonymity, etc.). Participants could take part only if they signed the informed consent form and were at least 18 years of age. The average time for completion was approximately 15 min.

#### 2.1.4. Statistical Analyses

The dimensionality and construct validity were calculated via exploratory factor analysis (EFA) of the main components. The final solution was based on the following criteria: appropriate number of dimensions according to the theoretical background, > 50% of the variance explained, and item loadings of > 0.400. Inter-factor correlations were analyzed, and descriptive statistics were calculated (average and standard deviation). Internal consistency was analyzed with Cronbach’s alpha.

### 2.2. Results

#### 2.2.1. Construct Validity

The first EFA resulted in a nine-factor model that explained 67.2% of the variance. Items with loadings below 0.400 were subsequently removed, and the EFA was repeated until a final model that met the abovementioned quality criteria was obtained. The final model consisted of 18 items distributed among five factors that explained 62.4% of the total variance (Table 1). These factors were named based on the theoretical dimensions proposed: (D1) identification, (D2) incorporation, (D3) reverberation, (D4) separation, and (D5) projection.

The descriptive statistics and inter-factorial correlations among the five dimensions are provided in Table 2. The results showed a positive relation between D1, identification with D3, reverberation, and D5, projection, but not with D2, incorporation or D4, separation. D2, incorporation did not show correlations with the other factors. D3, reverberation and D4, separation were significantly correlated. Finally, D5, projection showed significant positive correlations with D3, reverberation and D2, separation.

#### 2.2.2. Internal Consistency

Cronbach’s alpha was used to determine the internal consistency (reliability). Factor reliability varied from acceptable to good [53]: D1, identification, α = 0.70; D2, incorporation, α = 0.72; D3, reverberation, α = 0.77; D4, separation, α = 0.80; and D5, projection = α = 76. The global reliability of the scale was α = 0.78.

## 3. Study 2

Once the preliminary version of the Empathy Management Scale (EMS) containing 18 items had been obtained, Study 2 was necessary to test the five-dimensional model of Study 1. The convergent validity was also explored.

### 3.1. Method and Materials

#### 3.1.1. Participants

In total, 480 participants from Andalucía (Spain) were selected by controlled purposive sampling (67.3% female and 32.7% male). They ranged between 18 and 60 years of age (M = 27.8, SD = 11.8). Regarding formal education, most participants were undergraduates (81.4%). Of these, 49.4% were studying degrees in the field of humanities and health sciences (e.g., nursing) and 50.6% studied social and legal sciences (e.g., business administration and management). The remaining participants were distributed as follows: compulsory secondary education, 8.1%; primary education, 4.7%; A levels, 3.4%; unfinished primary education, 1.8%; and post-graduate studies, 0.7%.

#### 3.1.2. Instruments

In order to study the convergent validity of the EMS, the following instruments were used:

- EPE [38]. This scale comprises 31 items grouped into five subscales. Three scales were selected due to their relevance to our research goal. (a) Feelings: This subscale assesses the ability to detect others’ feelings, experience the same intensity and clarity of the emotions of others, and project emotions onto others (α = 0.79). (b) “Apata” characteristics: This subscale measures the difficulty to feel or experience others’ emotions (α = 0.79). (c) Separation and reverberation: This subscale concerns the influence of others’ emotions and the difficulty of empathic persons to separate themselves from these emotions (α = 0.69).
- IRI [10]; Spanish adaptation [12]. This instrument contains 28 items in four dimensions. The reliability for the four subscales were as follows: perspective taking, α = 0.66; fantasy, α = 0.62; empathic concern, α = 0.63; and personal discomfort, α = 0.68.
- Trait Meta-Mood Scale (TMMS-24) [47]; Spanish adaptation [11]. This scale measures individuals’ knowledge about their own emotional abilities (perceived EI). It comprises three dimensions: (a) emotional attention or the ability to feel and express emotions appropriately (α = 0.79), (b) emotional clarity or the ability to understand our own emotional states (α = 0.78), and (c) emotional repair or the ability to regulate our own emotional states appropriately (α = 0.86).
- Questions about sociodemographic variables were also included in the instrument (sex, age, and formal education).

#### 3.1.3. Procedure

The same procedure as Study 1 was carried out. The average time for completion was approximately 30 min.

#### 3.1.4. Statistical Analyses

Construct validity was obtained by a confirmatory factor analysis (CFA). The convergent validity was calculated by analyzing the relations (bivariate correlations) of the variables assessed by the instruments: empathy management (using the EMS), empathic personality (using the EPE), multidimensional empathy (using the IRI), and EI (using the TMMS-24). Finally, in order to obtain other indices of validity of the construct, individual differences in empathy management according to socio-demographics (sex, age, and field of knowledge (higher education degree)) were determined.

### 3.2. Results

#### 3.2.1. Construct Validity

A CFA approach was used to test competing models of the internal structure of the EMS. Two measurement models were tested: (1) a one-factor model including all 18 items and (2) a five-factor model including the identification factor (three items), incorporation factor (three items), reverberation factor (four items), separation factor (four items), and projection factor (four items).

The fit of the models was judged based on the guidelines provided by Hair et al. [54] for samples larger than 250 participants and instruments using between 12 and 30 items. The models were considered to fit the data if the CFI was > 0.92 and the SRMR was < 0.08, or the RMSEA was < 0.07. Only one of the tested models had an acceptable fit to the data, so measurement invariance by sex and age groups was analyzed only for that model based on a forward approach [55]. The five-factor model suggested by Study 1 showed a robust goodness of fit, χ^2^ = 240.5, *p* < 0.001; RMSR = 0.05. The fit indices calculated were satisfactory: NNFI = 0.89, CFI = 0.90, RMSEA = 0.04. The final instrument in Spanish is provided in the Appendix A (Table A1).

#### 3.2.2. Internal Consistency

The reliability indices varied from acceptable to good [53]: D1, identification, α = 0.73; D2, incorporation, α = 0.72; D3, reverberation, α = 0.76; D4, separation, α = 0.79; and D5, projection, α = 76. The global reliability of the scale was α = 0.75.

#### 3.2.3. Convergent Validity

Bivariate relations of the EMS with the total scores of the other instruments (the EPE, IRI, and TMMS-24) were significant (r = 0.487, *p* < 0.001; r = 0.483, *p* < 0.001; and r = 0.304, *p* < 0.001, respectively). There was convergence between the variables analyzed, where, as the ability to manage empathy increased, so too did the levels of empathy and EI.

In order to better understand this convergence, correlations of the instruments’ subscales were calculated (Table 3). D1, identification showed a significant positive relation with the feelings and separation subscales of the EPE instrument, and a negative relation with the “apata” characteristics. Focusing on EI, of the three dimensions evaluated by the TMMS-24, only clarity (awareness of one’s emotional states) achieved a positive correlation. Regarding the IRI, all relations were significant; the direction was positive for the dimensions of perspective taking, fantasy, and concern, and negative for the dimension of discomfort.

D2, incorporation showed correlations with the feelings and separation subscale of the EPE, inverse correlations with the attention and emotional regulation TMMS-24 subscales, and, finally, with the fantasy, concern, and discomfort subscales of the IRI.

D3, reverberation and D4, separation showed significant relations with the feelings (EPE) subscale and the TMMS dimensions. D3, likewise, showed a positive relationship with the separation subscale, and an inverse one with the “apata” subscale of the EPE. Finally, D5, projection was related to the three dimensions of EI. This dimension of empathy management was also positively related with the feelings subscale, and inversely with “apatas” (EPE). Regarding the IRI, D5 showed a statistically significant relationship with perspective taking and concern.

#### 3.2.4. Other Evidence of Construct Validity: Study of Sociodemographic Variables

Bivariate correlation analysis of the five dimensions of the EMS and age revealed a significant relationship between age and D1 and D2: as age increased, the initial stage of empathy, that is, identification, decreased (r = −0.206, *p* < 0.001), while incorporation increased (r = 0.141, *p* < 0.001).

Before carrying out the independent sample *t*-test, whether the variables met the assumption of normality was determined using the Kolmogorov−Smirnov test. All variables assessed (from D1 to D5) were significant at *p* > 0.05, i.e., were normally distributed. The mean comparison of participants’ sex (two: men, women) showed significant differences in D1, identification and D2, incorporation (t(475) = −3.35, *p* = 0.001). Women showed higher scores in both dimensions (identification, M_woman_ = 4.12, SD = 0.82, M_man_ = 3.84, SD = 0.91; incorporation, M_woman_ = 2.89, SD = 0.90, M_man_ = 2.58, SD = 0.88).

Regarding the field of study of the undergraduate participants (humanities and health sciences vs. social and law sciences), differences in scores were found only for D1, identification (t(467) = −4.00, *p* < 0.001). The humanities and health sciences students showed significantly higher scores in this dimension (M_Hum-health_ = 4.38, SD = 0.62, M_Soc-law_ = 3.89, SD = 0.81).

## 4. Discussion

The main goal of this study was to create and validate an instrument (the EMS) to measure the ability to manage empathy. The analyses carried out successfully validated the EMS, which comprises 18 items grouped into five dimensions: identification, incorporation, reverberation, separation, and projection. The psychometric fit of the scale was optimal, with appropriate validity and reliability indices.

The inter-factor (D1 to D5) and inter-construct (management of empathy, level of empathy, and EI) statistical relations showed similarities with multidimensional models of empathy [10,21], Reik’s theory [9], and current neuropsychological approaches [7].

The EMS dimensions describe empathic processes that are mainly activated in a sequential manner, thus revealing the inter-connections of the underlying mechanisms of empathy. The dimensional structure of the scale maintains the original sequence suggested by Reik (1948), and is in line with the subsequent reviews of Repetto (1977, 2009). Identification is confirmed as a fundamental variable in the empathy process: in the present study, it had by far the highest scores. However, contrary to what Repetto posited, identifying people’s emotions does not correlate nor overlap with incorporation. They seem, in fact, to be two different constructs. These results are in line with neuroanatomic and functional research on empathy. Emotional aspects of empathy (i.e., sharing emotional states) relate to mirror neurons, while cognitive aspects (i.e., perspective taking and theory of mind (ToM)) mainly relate to high-level cognitive processes [56,57]. In this sense, except for incorporation (D2), the EMS dimensions seem to fit within ToM, albeit with nuances in terms of emotional processing. Identification (D1) implies understanding, and reverberation (D3) reflects being aware. Both factors, which are correlated, share processes regulated by executive brain functions. Separation (D4) and projection (D5), which are significantly related to each other, require conscious management and strategic use of abilities to prepare for a difficult situation and to understand how to appease someone, respectively. Projection was added to Reik’s empathy process, and is considered as the final stage. This factor is particularly relevant in the professional context. Whether it is a conscious strategy or an inherent ability naturally transmitted, it can only take place if the other person’s state has been identified and preparation to manage the situation (separation and reverberation) has been undertaken.

As previously mentioned, the stage of identification is mainly cognitive, since perceiving and experiencing someone else’s feelings requires understanding their emotions and adopting their perspective. This description is similar to the cognitive processes posited by Davis in his multidimensional model [6,10]. In fact, significant correlations between the identification and perspective-taking and fantasy subscales of the IRI were found. The incorporation stage shares some features with empathic concern and personal discomfort in terms of emotional processes. The ability to project (projection) was also confirmed to only occur if the other’s point of view has been understood (perspective-taking) and feelings of compassion, concern, and care for their unease are felt (empathic concern).

Empathy management and EI were related. The dimensions of the TMMS pertain to expressing emotions (attention) and understanding (clarity) and regulating them (repair). These processes relate positively with factors from the EMS linked to active control processes and empathy management (reverberation or being aware, separation or proactivity/preparation, and projection or transmission of emotions). The negative relation between reparation (TMMS) and incorporation (EPE) shows that as emotional contagion increases, emotional regulation decreases. These results add nuance to those of studies showing relations between the dimensions of the TMMS and empathy [58].

Regarding the EPE [38], the analysis revealed significant relations between the feelings subscale and the five dimensions of the EMS, and between the separation subscale and D1, D2, and D3 (EMS). Both instruments share a theoretical background, as reflected in the results. The novelest findings relate to the concept of “apata”, evaluated through the EPE: people with low empathy (“apatas”) are defined not only by low identification (a result already found in previous literature), but also by worse management of empathy, that is, lower reverberation and projection.

Overall, the abovementioned results point to the following tendency in the management of empathy. As a professional moves through the stages linked to better management of empathy (separation and projection), the other’s perspective is mentally adopted (identification), while the discomfort generated by the negative experience of others is left behind (adaptive incorporation, reverberation, and restoration of balance). Therefore, for a person to be considered as empathic and optimally regulating all of the processes thereof, high levels of identification, low to moderate levels of incorporation, and high levels of reverberation, separation, and projection are needed. Such a professional (nurse, psychologist, etc.) would have the ability to follow-up in a reflexive way on his own emotions and those of others, and to distinguish the origin of the emotions. Ultimately, they would be aware of the processes involved in managing empathy effectively. Conversely, high levels of incorporation and low levels of reverberation would make it impossible for the person to move through the various stages and finally obtain a well-balanced empathic process. This approach could explain phenomena such as professional exhaustion due to excessive compassion [1,15].

According to the analysis of EMS scores according to sociodemographic variables such as sex, age, and field of study, the results matched those of previous research. Regarding sex, a considerable number of studies showed differences of emotional empathy between men and women. Women experience emotional empathy to a higher degree than men (both positive and negative) [59], as well as higher compassion, concern, and care in the face of others’ discomfort [12,58]. These studies also showed that women are more capable of putting themselves into others’ shoes and understanding their situation [12,58]. Women’s higher levels of identification and incorporation in the present study reflect both tendencies. Some approaches consider that men tend more towards instrumental actions in the context of empathy [60]. However, such differences were not found in most instrumental dimensions of the EMS in this study: women and men had similar reverberation, separation, and projection abilities. In line with this, recent studies have questioned the existence of such rigid differences in empathy between men and women. Sex differences could result from the interaction of multiple factors, such as social learning, genetic predisposition, evolutionary underpinnings, and interpersonal styles [61,62]. The women in the present study scored higher on the emotional components; as these are more dependent on neurochemical processes, it is intuitive for sex differences to exist. However, as a consequence of sociocultural changes (i.e., a more egalitarian society), men are increasingly using their empathic capabilities; thus, sex differences in this construct are decreasing.

The relationship between age and empathy has been widely studied, and there seems to be agreement that the latter increases progressively from childhood until the teenage years, particularly in terms of emotional regulation [63]. Studies on empathy in young and older adults are less common, and the results less conclusive. Some works on this topic concluded that age gradually increases the ability to place oneself in the shoes of others. In addition, negative emotional reactions (personal discomfort) transform into sympathy and compassion towards others with age [64,65]. However, some other research has suggested the contrary, i.e., that cognitive empathy decreases with age and older adults experience higher levels of emotional distress and reactivity in the face of someone else’s discomfort [66]. The results from the present study are in line with the latter.

Finally, the students studying degrees such as psychology and nursing in this study had a greater ability to identify and understand emotional states compared to law and business management and administration students. This result could be explained in various ways. Differences in D1 could be due to differences in individual traits, such as cognitive/motivational determinants or features, such as values or empathy. Alternatively, the differences could be the result of specific training and the core competencies of each university degree. The scarce literature on this matter makes it difficult to compare data, and the few results that are available did not find differences in empathy according to the type of degree studied.

This study has certain limitations, potentially including the number of participants, which was relatively close to the minimum advised for this type of research. Likewise, the intentional sampling limits our ability to generalize the results. In addition, the use of college undergraduates may reduce the generalizability of the results, although they are somewhat similar to professionals. Because empathy directly involves other people and occurs in social interactions, it can be measured from an external perspective [67]; thus, it would have been instructive to address external validity, although this could be a target for future research. Further studies could include objective measures (e.g., the Situational Test of Emotional Management, STEM [68]; Diagnostic Assessment of Non Verbal Affect-Adult Facial Expressions, DANVA2-AF [69]; and Diagnostic Assessment of Non Verbal Affect-Adult Paralanguage, DANVA2-AP [70]) and ratings of others. Moreover, the use of the EME in a broader range of participants (i.e., healthcare professionals) would enhance the generalizability of the results beyond students. Other avenues of research include establishing causal models for the studied variables, as well as implementing the EMS in clinical contexts.

## 5. Conclusions

The EMS assumes that empathy is a multidimensional construct, and describes empathy processes based on cognitive and emotional dimensions. The sequential stages of the model reflect the inter-dependence of bottom-up processes, or direct perception processes, and top-down processes that imply regulation and control [7]. The scale makes an important contribution by measuring a wide range of behaviors that capture the ability to manage empathy appropriately, namely reverberation, separation, and projection. It is a particularly convenient instrument for use in professions where empathy is a necessary skill for ensuring positive interactions with patients/users, including for training purposes. Implementation of the EMS would deepen analyses of the disadvantages of feeling too much empathy in the absence of the skills needed to manage an emotional contagion and overload, and could facilitate the design of an intervention to restore the balance between feeling empathy and knowing how to manage it.

## Figures and Tables

**Table 1:** EFA of the five Empathy Management Scale (EMS) factors. Item loadings for each dimension are shown.

Item	D1	D2	D3	D4	D5
17 (8 *) People’s negative emotions are so easily passed on to me that they affect me as if they were my own.		0.817			
26 (13) When I am next to someone who is upset, I end up feeling the same way.		0.832			
10 (6r **) When I am next to someone who is upset, their emotions do not transfer to me.		0.713			
4 (4) When I know I will be exposed to an emotionally negative and intense situation, I prepare to distance myself from the situation.				0.812	
1 (1) Before a difficult situation, I use some techniques to face the emotions to come, such as relaxation.				0.807	
18 (17r **) I avoid situations involving negative emotions because I don’t know how to get rid of them.				0.798	
19 (18) I know how to separate myself from others’ feelings.				0.736	
2 (2r) It is difficult for me to identify which negatives emotions are my own and which are the other person’s.			0.802		
3 (3) I am able to determine if the emotions I experience are my own or the other person’s.			0.800		
23 (11) When I am with someone, I can identify which part of what I am feeling is caused by that person.			0.701		
5 (5) When it comes to positive emotions, I can easily identify which have been passed on to me by the other person and which are my own.			0.676		
21 (10r) When someone explains to me how they feel, it is difficult for me to understand it.	0.771				
28 (15r) I struggle to understand how another person is feeling.	0.772				
20 (9r) I tend not to understand how someone is feeling by their non-verbal communication.	0.733				
13 (7) I know how to appease someone.					0.748
25 (12) If someone is feeling sad, I pass my joy on to him/her.					0.750
30 (16) When someone near me is feeling anxious, I don’t know how but I am able to calm him/her.					0.671
27 (14r) It is difficult for me to create positive emotions in a difficult situation.					0.652

* Final sequence of items after EFA and CFA. ** Reverse items (sub-index ‘r’).

**Table 2:** Descriptive statistics and inter-factor correlations.

EME Dimensions	Correlations					
	M (SD)	D1	D2	D3	D4	D5
D1Idt	4.00 (0.92)	1				
D2Inc	2.67 (0.93)	−0.032	1			
D3Rev	3.52 (0.86)	0.112 **	0.033	1		
D4Sep	3.07 (0.88)	−0.056	0.053	0.293 **	1	
D5Proj	3.17 (0.73)	0.137 **	0.049	0.267 **	0.375 **	1

** The correlation is significant at the 0.01 level (bilateral).

**Table 3:** Correlations among EMS, EPE, TMMS-24, and IRI subscales.

Subscales	1	2	3	4	5	6	7	8	9	10	11	12	13	14	15
1. EMSiden	1														
2. EMSinc	−0.052	1													
3. EMSrev	0.125 **	−0.024	1												
4. EMSsep	−0.027	−0.032	0.243 **	1											
5. EMEproj	0.126 **	−0.027	0.255 **	0.343 **	1										
6. EPEfeel	0.342 **	0.250 **	0.126 **	0.264 **	0.392 **	1									
7. EPEsep	0.114 *	0.333 **	0.099 *	−0.023	0.074	0.385 **	1								
8. EPEapat	−0.334 **	0.039	−0.145 **	−0.022	−0.140 **	−0.066	−0.080	1							
9. TMMSaten	0.058	0.233 **	0.095 *	0.140 **	0.143 **	0.299 **	0.241 **	0.151 **	1						
10. TMMSclar	0.185 **	−0.075	0.103 *	0.377 **	0.308 **	0.244 **	−0.022	0.153 **	0.130 **	1					
11. TMMSreg	0.087	−0.180 **	0.301 **	0.282 **	0.407 **	0.157 **	−0.017	0.120 **	0.043	0.301 **	1				
12. IRIpers	0.340 **	0.033	0.171 **	0.165 **	0.287 **	0.397 **	0.206 **	0.171 **	0.218 **	0.248 **	0.281 **	1			
13. IRIfant	0.207 **	0.213 **	−0.003	−0.007	−0.027	0.237 **	0.193 **	0.185 **	0.275 **	−0.006	−0.084	0.137 **	1		
14. IRIconcer	0.308 **	0.295 **	0.048	0.079	0.168 **	0.406 **	0.468 **	0.141 **	0.378 **	0.086	0.042	0.429 **	0.241 **	1	
15. IRIdisc	−0.188 **	0.355 **	−0.056	−0.135	−0.213	−0.023	0.200 **	−0.023	0.221 **	−0.256 **	−0.249	−0.120 **	0.186 **	0.134 **	1

** The correlation is significant at the 0.01 level (bilateral). * The correlation is significant at the 0.05 level (bilateral).

## Data Availability

The data that support the findings of this study are available from the corresponding author (M.A.) upon reasonable request.

## Appendix A

**Table A1:** Escala de Manejo de Empatía (EME) in Spanish.

(1) Previamente a una situación difícil, empleo técnicas para afrontar las emociones que me esperan, como la relajación.	1	2	3	4	5
(2) Es difícil para mí darme cuenta de qué emociones negativas son mías y cuáles provienen de otra persona.	1	2	3	4	5
(3) Soy capaz de darme cuenta de si los sentimientos que experimento son míos o provienen de otra persona.	1	2	3	4	5
(4) Cuando sé que me voy a exponer a una situación emocionalmente negativa e intensa me preparo de alguna manera para distanciarme de esa situación.	1	2	3	4	5
(5) Cuando se trata de emociones positivas me doy cuenta con facilidad de cuáles me las ha contagiado otra persona y cuáles he generado yo.	1	2	3	4	5
(6) Aunque esté con otra persona que se siente mal, no me contagio de su emoción.	1	2	3	4	5
(7) Sé tranquilizar a las personas mediante mi contacto.	1	2	3	4	5
(8) Me contagio tan fácilmente de las emociones negativas de los demás que me afectan como si fueran mías.	1	2	3	4	5
(9) No suelo saber cómo se siente alguien por su comunicación no verbal.	1	2	3	4	5
(10) Cuando otra persona me explica cómo se siente, me resulta difícil entenderla.	1	2	3	4	5
(11) Cuando estoy con otra persona me doy cuenta de qué parte de lo que siento me está generando ella.	1	2	3	4	5
(12) Si alguien está triste, le contagio la alegría.	1	2	3	4	5
(13) Cuando estoy con otra persona que se siente mal termino sintiéndome igual de mal que ella.	1	2	3	4	5
(14) Me resulta difícil;cil generar emociones positivas en una situación difícil.	1	2	3	4	5
(15) Me cuesta trabajo entender cómo se siente otra persona.	1	2	3	4	5
(16) Cuando tengo cerca a una persona que está nerviosa, puedo no saber cómo lo hago, pero la tranquilizo.	1	2	3	4	5
(17) Evito situaciones que impliquen emociones negativas porque no sé cómo deshacerme de ellas.	1	2	3	4	5
(18) Sé cómo separar mis sentimientos de los de los demás.	1	2	3	4	5
1 = totalmente en desacuerdo, 2 = en desacuerdo, 3 = ni en desacuerdo, ni de acuerdo, 4 = de acuerdo, 5 = totalmente de acuerdo					
